# Cerebral Arteriovenous Malformation Recurrence After Complete Surgical Excision in an Adult: Case Report and Review of the Literature

**DOI:** 10.7759/cureus.15366

**Published:** 2021-06-01

**Authors:** Daniel Loh, Vincent Ng

**Affiliations:** 1 Neurosurgery, National Neuroscience Institute, Singapore, SGP

**Keywords:** arteriovenous malformations, recurrence, adult, complete excision, follow-up

## Abstract

Angiographically confirmed complete surgical excision of brain arteriovenous malformations (bAVMs) is conventionally considered curative. Recurrence in adults is rarely encountered; only 18 cases have been reported in the English literature over the past 30 years. The potential for recurrence and consequent need for routine long-term follow-up are important considerations in the management of these lesions. We report a case of a 23-year-old female with a recurrent bAVM discovered incidentally on routine imaging three years after complete surgical excision. We review the existing literature and discuss the options for surveillance and management.

## Introduction

Brain arteriovenous malformations (bAVMs) are congenital anomalies of dysplastic blood vessels with direct connections between arteries and veins without intervening capillaries which form a tangle of abnormal dilated channels called a nidus. Most of these lesions are discovered incidentally during imaging for other indications; symptomatic bAVMs most commonly present with haemorrhage and seizures [1-3]. bAVM recurrence after angiographically confirmed complete surgical resection is an uncommon but well recognized phenomenon in children, with an average rate of recurrence of 9.5% [4] (ranging from 2.8% - 16%) [5-11], occurring from several months to 16 years after initial resection [12]. Recurrence is much rarer in adults, with only 18 cases reported in the English literature in the last 30 years. We report a case of bAVM recurrence in a patient who was 23 years old at the time of first presentation who had asymptomatic recurrence three years later.

## Case presentation

**Figure 1:**
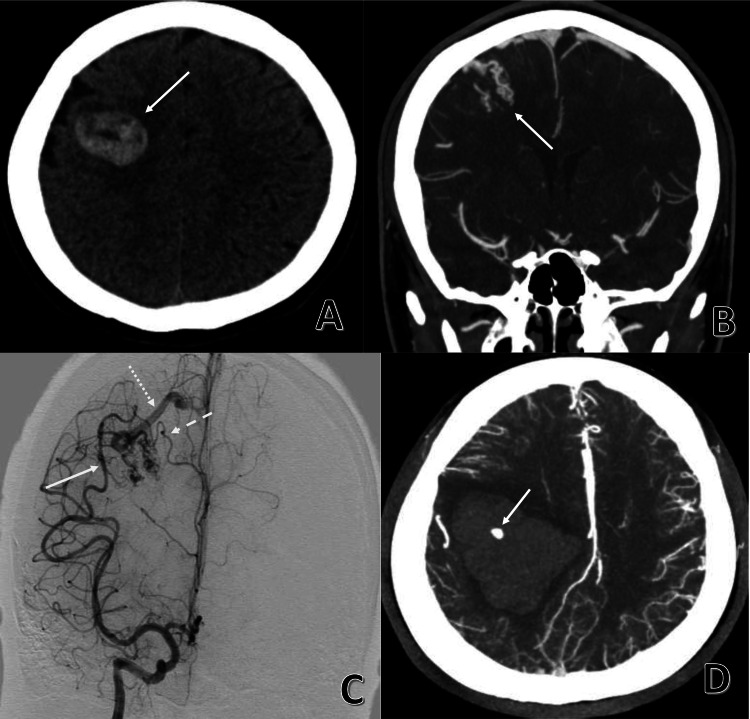
Computed tomography (A, B and D) and catheter angiogram (C) demonstrating the Spetzler-Martin grade 1 brain arteriovenous malformation and the associated intraparenchymal haematoma (A) Computed tomography showing a right frontal haematoma (solid arrow). (B) Computed tomography angiogram revealing a right frontal brain arteriovenous malformation (solid arrow). (C) Digital subtraction angiogram demonstrating the brain arteriovenous malformation fed by branches of the middle cerebral artery (solid arrow) and anterior cerebral artery (dashed arrow) with superficial drainage into the superior sagittal sinus (dotted arrow). (D) Urgent computed tomography after clinical deterioration showing haematoma expansion, intra-ventricular extension and evidence of contrast extravasation suggestive of active bleeding (solid arrow).

A 23-year-old right-handed lady with no medical history presented in April 2017 to a district general hospital with acute onset left brachiofacial hemiparesis. A computed tomography (CT) scan of the brain showed a right frontal intraparenchymal haematoma (Figure 1A) and CT angiogram (CTA) revealed a right frontal AVM (Figure 1B). She was transferred to our hospital for further management and a digital subtraction angiogram (DSA) was performed on the same day which showed a Spetzler-Martin grade 1 bAVM with intranidal aneurysms in the right posterior frontal lobe measuring 2.4cm x 1.7cm x 0.8cm supplied by branches of the right middle and anterior cerebral arteries draining into the superior sagittal sinus (Figure 1C). She was neurologically stable and planned for delayed surgical resection to await partial clot liquefaction. However, she deteriorated three days later with a drop in her Glasgow Coma Scale (GCS) score from 15 to E3V2M4. An urgent CTA showed marked increase in size of the haematoma with extensive intra-ventricular extension and a 6mm CT-spot sign suggesting active haemorrhage (Figure 1D).

**Figure 2:**
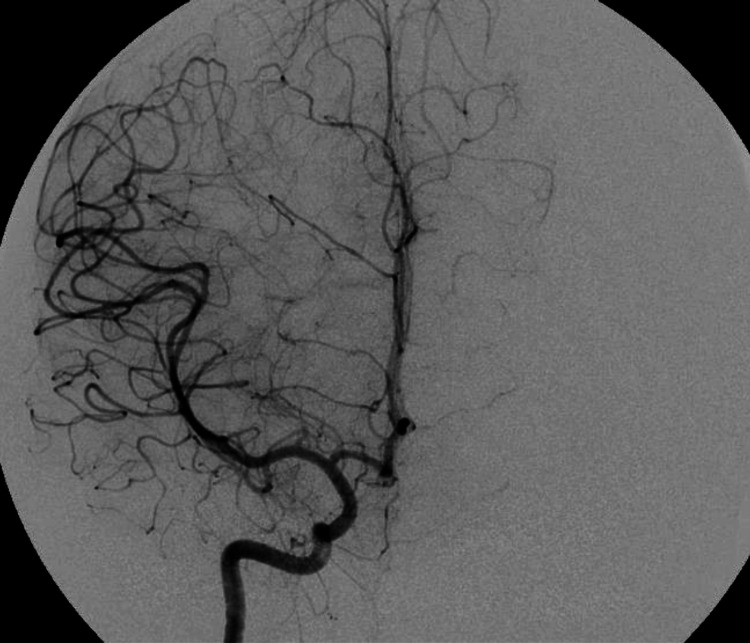
Immediate post-operative digital subtraction angiogram showing no residual brain arteriovenous malformation

She was taken to theatre emergently for decompressive craniectomy, evacuation of the haematoma and excision of the bAVM. Intra-operative indocyanine green (ICG) angiography revealed two main arterial feeders on the cortical surface and one main draining vein to the superior sagittal sinus. The bAVM was excised en-bloc and post-excision ICG showed no remnant which was confirmed on immediate post-operative DSA (Figure 2).

**Figure 3:**
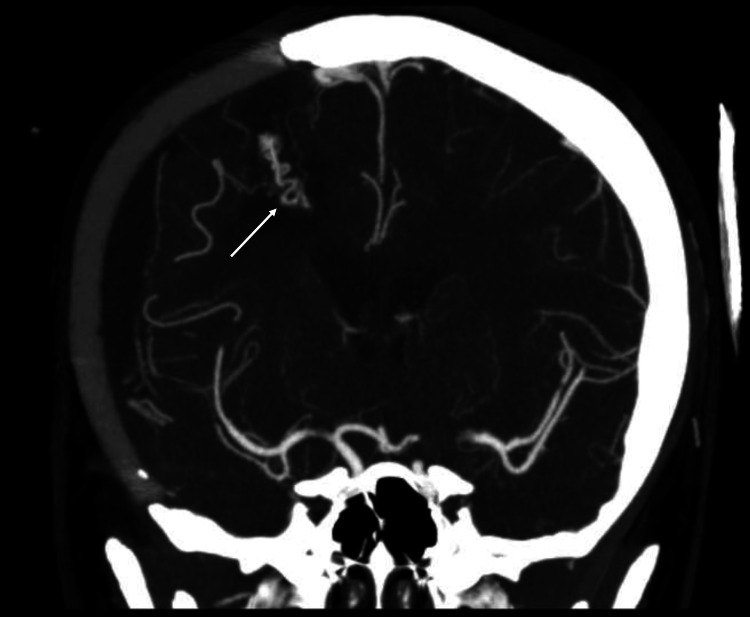
Routine computed tomography angiogram revealing recurrence of the brain arteriovenous malformation (solid arrow) in the same location as the one excised three years ago

She was transferred to our rehabilitation centre 19 days after surgery. While undergoing rehabilitation she had a generalised seizure attributed to scar epilepsy which was controlled with levetiracetam. She recovered well; her Functional Independence Measure (FIM) score improved from 28 to 108, and she had a cranioplasty implant four months later. She remained clinically well on six-monthly follow-up apart from another seizure in June 2018. A non-enhanced CT showed no haemorrhage and she had no further seizures after the dose of levetiracetam was increased. A routine CTA in August 2020 showed partial recanalization of the same bAVM nidus, now supplied via tiny right middle cerebral artery (MCA) (and possibly anterior cerebral artery [ACA]) cortical branch(es) with venous drainage towards the superior sagittal sinus (Figure 3).

**Figure 4:**
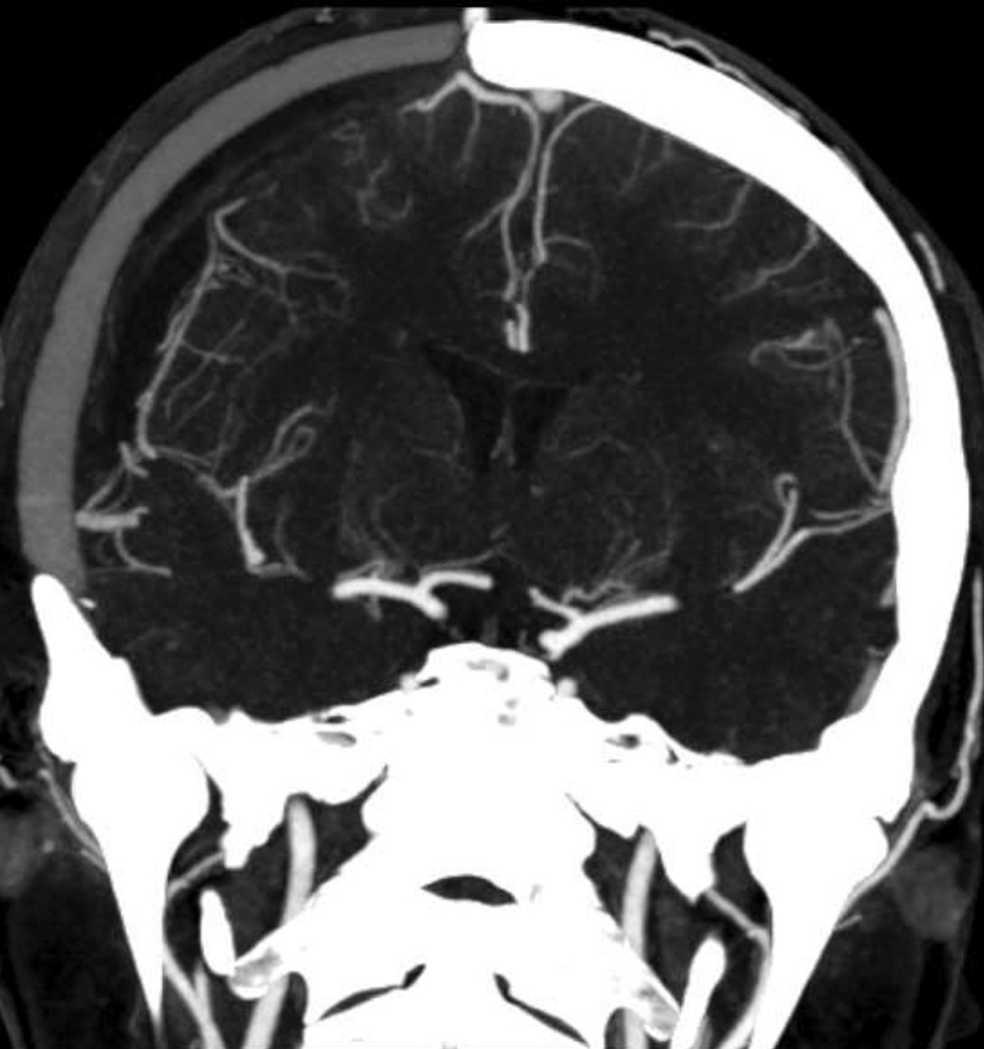
Post-operative day 1 computed tomography angiogram showing no residual brain arteriovenous malformation

In light of her age and good functional status, repeat surgical resection was recommended although the option of stereotactic radiosurgery was also offered. After consulting with a radiosurgeon, she decided to proceed with surgical resection. A stereotactic CTA repeated in February 2021 showed stable size of the bAVM. She underwent resection of the recurrent AVM in March 2021 with stereotactic navigation via CTA. Intra-operatively, small cortical feeders were seen which were taken circumferentially; no large draining vein was identified. Post-operative day 1 CTA showed no remnant bAVM (Figure 4). She had an uneventful post-operative course; her function remained at baseline and she was discharged well one week after surgery with a plan for delayed DSA after a few months.

## Discussion

bAVM recurrence in adults after angiographically demonstrated complete excision is a rare phenomenon. Only sporadic case reports/series exist; just 18 cases have been reported in the English literature over the last 30 years (Table 1) [9, 13-15, 16-24]. Age at initial presentation ranged from 19 to 51, recurrence was observed from several months to 16 years after resection with all except one discovered within 10 years. No predilection for gender or bAVM location is apparent. Patients in earlier reports presented symptomatically with haemorrhage or seizures while those reported more recently were discovered on routine imaging during follow-up. Most cases (10/18) were managed by surgical resection. In our case, the isolated seizure in June 2018 may have represented clinical manifestation of her bAVM recurrence which was discovered on routine follow-up imaging three years later and treated by repeat surgical excision.

**Table 1:** Summary of reported cases of AVM recurrence after complete excision in adult patients AVM: Arteriovenous malformation; SRS: Stereotactic radiosurgery.

Authors, year	Number of cases	Age at first presentation	Gender	Duration till recurrence (years)	Location of AVM	Discovery	Management
Fuwa et al., 1988 [16]	1	23	F	5	Right temporal	Routine imaging	Surgical resection
Gabriel et al., 1996 [17]	1	19	M	9	Left frontotemporal	Seizure	Surgical resection
Pellettieri et al., 1997 [15]	1	29	F	15	Right frontal	Seizure	Not mentioned
Hashimoto & Nozaki, 1999 [18]	2	24, 23	M, F	8, 6	Left basal ganglia, Right frontal	Haemorrhage	Not mentioned
Hino et al., 1999 [19]	1	28	M	4	Right frontal	Haemorrhage	SRS
Santoro et al., 2000 [20]	1	24	M	6	Right temporal	Seizure	Surgical resection
Codd et al., 2008 [14]	1	26	F	7	Left occipital	Haemorrhage	Surgical resection
Musluman et al., 2011 [21]	1	35	F	5	Left occipital	Seizure	Surgical resection
Wostrack et al., 2011 [22]	1	30	F	2.5	Left parietal	Routine imaging	Surgical resection
Morgan et al., 2012 [9]	3	42, 26, 28	F, M, F	3, 1.5, 2.5	Left temporal, Midbrain/thalamus, Cingulate	Routine imaging all 3	Surgical resection all 3
Weingarten et al., 2012 [23]	1	20s	M	0.5	Left frontoparietal	Routine imaging	Embolisation + SRS
Ivanov et al., 2016 [13]	3	36, 30, 31	F, F, M	3, 1, 3	Left splenium, Right splenium, Left frontal	Routine imaging first 2 Headache	SRS all 3
Marutani et al., 2020 [24]	1	51	F	5	Right temporal	Haemorrhage	Surgical resection

Identified risk factors for bAVM recurrence include initial presentation with haemorrhage [8], the presence of deep venous drainage [9], a diffuse nidus [11] and treatment with preoperative embolization [13].

These factors are largely consistent with the postulated mechanisms behind bAVM recurrence. The first is that recurrence actually represents residual nidus that was angiographically occult due to obscuration from vessel spasm, temporary thrombosis or mass effect from adjacent haematoma or cerebral oedema during the immediate post-operative period. Subsequent resolution would then result in recanalisation of the remnant shunt [10,14]. Another possibility is the concept of “hidden compartments” which are unfilled regions of the AVM contiguous with or adjacent to the active nidus because of low or absent flow from internal steal that develop from the change in haemodynamics following resection [15].

Recurrence can also be a result of neoangiogenesis and de novo bAVM formation, which would readily explain the far greater incidence of bAVM recurrence in the paediatric population. Elevated levels of vascular endothelial growth factor (VEGF) have been found in adult samples [25], and higher rates of elevated VEGF expression have been demonstrated in paediatric cases with bAVM recurrence compared to paediatric and adult cases without recurrence [26]. Increased expression of other angiogenetic factors such as KRAS mutations [27], phosphorylated extracellular signal-regulated kinase (pERK) and CD105 (endoglin) [28] has also been reported. De novo bAVM formation after venous sinus thrombosis has also been observed, with impaired venous outflow leading to congestion, parenchymal ischaemia and hypoxia and increased angiogenesis as the proposed mechanism [29].

There is little evidence to guide the duration, schedule and modality of follow-up imaging in adult patients after complete surgical resection; there is no consensus even in the paediatric literature [30,31]. Based on suggested follow-up in children [31] and from the adult cases thus far, follow-up for 10 years, with routine imaging at 1 year, 5 years, 10 years and for any new or progressive neurological signs and symptoms, would be reasonable. Although DSA is still the gold standard modality and was utilized to detect recurrence in all reported cases, a 2019 study found that contrast-enhanced magnetic resonance imaging and angiography (MRI/MRA) was suitable for surveillance, with DSA subsequently used for confirmation [32].

The appropriate treatment strategy is similarly poorly established, and the risk of re-rupture of these recurrent bAVMs is unknown. Just over half the adult recurrences were treated by surgical excision, stereotactic radiosurgery (SRS) was the next most common option. A systematic review in 2018 that included paediatric cases found that surgery (59%) and SRS (37%) were the most common methods of treatment [4] and a recent consensus paper recommended that recurrent lesions be treated surgically [33]. Ultimately, decisions on treatment of these lesions should be reached via consensus between the patient and clinician after a comprehensive discussion of the risks and benefits. In this case, the existing cranioplasty posed an additional element of complexity to the consideration for repeat surgery.

## Conclusions

bAVM recurrence in adults after complete surgical resection is a rare but potentially important phenomenon. Routine follow-up imaging over an extended duration may be warranted, especially in cases with one or more risk factors for recurrence. New or progressive clinical manifestations should similarly trigger vascular imaging. Non-invasive imaging modalities may be a viable alternative to DSA for surveillance. Repeat surgical excision and SRS were the most common methods of treatment.
